# The ScinTwelve Study: Pharmacoscintigraphic Evaluation and Pharmacokinetic Assessment of a Vitamin B12 Sustained-Release Tablet in Healthy Human Subjects

**DOI:** 10.7759/cureus.114721

**Published:** 2026-08-18

**Authors:** Nency Vasoya, Ameet Soni, Nimesh Modi, Prakash A Jadhav, Lalit Tyagi, Nitin Sharma, Kushagra Khanna

**Affiliations:** 1 Department of Medical Affairs, Corona Remedies Limited, Ahmedabad, IND; 2 Department of Research &amp; Development, Corona Remedies Limited, Ahmedabad, IND; 3 Department of Pharmaceutics, Lloyd Institute of Management &amp; Technology, Greater Noida, IND; 4 Department of Pharmaceutics, Amity University, Noida, IND; 5 Faculty of Pharmaceutical Sciences, UCSI University, Kuala Lumpur, MYS

**Keywords:** drug delivery, gamma scintigraphy, gastric emptying, pharmacoscintigraphy, sustained-release tablet, vitamin b12

## Abstract

Background: Oral bioavailability of vitamin B12 is highly dependent on gastrointestinal transit and site-specific release, with optimal absorption occurring in the terminal ileum. Sustained-release formulations were designed to enhance targeted delivery and prolong systemic exposure.

Objective: This study aimed to evaluate the in vivo disintegration pattern, gastric emptying, and gastrointestinal transit of a vitamin B12 sustained-release tablet using pharmacoscintigraphy and to correlate these findings with systemic bioavailability.

Methods: An open-label, single-dose pharmacoscintigraphy study was conducted in six healthy adult male subjects. The investigational product (vitamin B12 1500 mcg sustained-release tablet) was radiolabeled with technetium-99m (^99m^Tc) and administered orally. Sequential gamma imaging was performed up to eight hours post dose to determine the gastric residence, intestinal arrival, intestinal residence, and colon arrival time. Serum vitamin B12 levels were measured at predefined intervals up to 48 hours.

Results: The upper placebo layer disintegrated rapidly in the stomach within 1.0 ± 0.0 minutes, with gastric emptying completed by 0.7 ± 0.2 hours. The lower sustained-release layer remained intact in the stomach and emptied at 120.00 ± 0.00 hours, followed by gradual intestinal disintegration between four and six hours. Colonic arrival occurred at 336.00 ± 53.67 hours. Serum vitamin B12 levels peaked at eight hours (296.00 ± 94.65 pg/mL), indicating delayed absorption corresponding to intestinal release. Sustained levels were maintained up to 48 hours, and no adverse events were reported.

Conclusion: The vitamin B12 sustained-release tablet exhibited effective biphasic release with targeted intestinal delivery and sustained bioavailability, confirming its design intent and potential for improved therapeutic efficacy.

## Introduction

Vitamin B12 (cobalamin) is an essential water-soluble vitamin that plays a pivotal role in hematopoiesis, neurological function, and DNA synthesis. It serves as a cofactor in key enzymatic reactions, including the conversion of homocysteine to methionine and methylmalonyl-CoA to succinyl-CoA, thereby contributing to cellular metabolism and myelin integrity. Deficiency of vitamin B12 is associated with a spectrum of clinical manifestations ranging from megaloblastic anemia to irreversible neurological complications such as peripheral neuropathy, cognitive impairment, and subacute combined degeneration of the spinal cord. The prevalence of vitamin B12 deficiency is particularly high among elderly populations, vegetarians, and patients with gastrointestinal disorders, including pernicious anemia, atrophic gastritis, and post-gastrectomy conditions [1].

Despite the widespread use of oral supplementation, the bioavailability of vitamin B12 is inherently limited by its complex and highly regulated absorption pathway. Following oral administration, vitamin B12 must first be released from the dosage form in the gastric environment, where it binds to salivary haptocorrin. Subsequently, in the duodenum, pancreatic enzymes facilitate its release from haptocorrin, enabling binding to intrinsic factor, a glycoprotein secreted by gastric parietal cells. The vitamin B12 intrinsic factor complex then travels to the terminal ileum, where it is absorbed via receptor-mediated endocytosis. This multi-step process imposes significant constraints on the efficiency of absorption, particularly in conditions where intrinsic factor secretion or ileal function is compromised. Moreover, passive diffusion accounts for only a negligible fraction of total absorption, further emphasizing the importance of targeted delivery to the ileum [2].

Conventional immediate-release oral formulations of vitamin B12 often fail to align drug release with this physiological absorption window. Rapid disintegration and dissolution in the stomach or proximal small intestine may result in suboptimal availability of free vitamin B12 at the distal ileum, thereby limiting systemic exposure. Additionally, variability in gastric emptying and gastrointestinal transit times can further contribute to inconsistent absorption profiles. These challenges highlight the need for advanced formulation strategies capable of modulating the spatial and temporal release of vitamin B12 within the gastrointestinal tract.

Sustained-release oral delivery systems have emerged as promising approaches to modulate the pharmacokinetic parameters. In this context, Sustained-release tablets of vitamin B12 are developed to release vitamin B12 slowly over several hours, rather than all at once. More consistent plasma levels of vitamin B12 throughout the day, which may help to avoid sharp peaks and troughs, which can occur with immediate-release formulations. It mimics the natural vitamin delivery rhythm of the body more closely than an immediate-release dose. However, the successful development of such complex delivery systems necessitates robust in vivo evaluation to confirm their performance. Traditional in vitro dissolution testing, although essential during formulation development, often lacks the ability to accurately predict in vivo behavior, particularly for formulations intended for site-specific delivery. Factors such as gastrointestinal motility, luminal pH variations, enzymatic activity, and fluid dynamics cannot be fully replicated in vitro. Consequently, there is a growing need for advanced methodologies that enable direct visualization of dosage form behavior within the human gastrointestinal tract [3-5].

Pharmacoscintigraphy, a non-invasive imaging technique based on gamma scintigraphy, has emerged as a powerful tool for evaluating the in vivo performance of oral drug delivery systems. This technique involves the incorporation of a gamma-emitting radionuclide, such as technetium-99m (^99m^Tc), into the dosage form, allowing real-time tracking of its transit, disintegration, and dispersion within the gastrointestinal tract. Pharmacoscintigraphy provides critical insights into key parameters such as gastric residence time, intestinal arrival, site of disintegration, and colonic transit. Importantly, when combined with pharmacokinetic measurements, this approach enables the correlation of physical dosage form behavior with systemic drug absorption, thereby offering a comprehensive understanding of formulation performance [6].

The vitamin B12 1500 µg sustained-release tablet investigated in the present study is a bilayered formulation containing a placebo immediate-release layer and a methylcobalamin sustained-release layer. The formulation has been specifically designed so that it delivers vitamin B12 continuously throughout the gastrointestinal tract. Vitamin B12 absorption is saturable at low doses (via intrinsic factor), but passive diffusion occurs at a rate of ~1% of dose, regardless of saturation. The sustained-release tablets are taken at high doses (e.g., 1500 µg), which can deliver vitamin B12 slowly, potentially increasing the fraction absorbed via passive diffusion over time. This may be useful in patients with impaired intrinsic factor function, like those with atrophic gastritis or gastric bypass, though less effective than injections in severe cases. Given the physiological importance of ileal absorption for vitamin B12, it is critical to confirm that the delayed-release component of the formulation reaches the distal intestine in an intact state and releases the drug in a controlled manner [4, 7, 8]. Pharmacoscintigraphy offers a direct and reliable means to validate this behavior in vivo. Furthermore, correlating the imaging data with serum vitamin B12 levels allows for a deeper understanding of the relationship between gastrointestinal transit, site of release, and systemic exposure. Therefore, the present study was designed to comprehensively evaluate the in vivo disintegration pattern, gastric emptying behavior, and gastrointestinal transit of the bilayered vitamin B12 sustained-release tablet using pharmacoscintigraphy in healthy adult subjects. In addition, the study aimed to assess the pharmacokinetics of vitamin B12 and establish a temporal correlation between imaging-derived parameters and pharmacokinetic outcomes. By integrating imaging and pharmacokinetic data, this investigation seeks to provide mechanistic evidence supporting the ability of the formulation to achieve site-specific delivery and sustained systemic exposure.

## Materials and methods

Study design

This investigation was designed as a prospective, single-center, open-label, single-dose pharmacoscintigraphic study to evaluate the in vivo disintegration behavior, gastric emptying, gastrointestinal transit, and pharmacokinetics of a bilayered vitamin B12 sustained-release oral tablet in six healthy adult human subjects. The study was conducted under post-marketing surveillance conditions to generate mechanistic evidence supporting formulation performance. The study followed a single-arm design with no comparator, as the primary objective was exploratory and focused on characterizing in vivo formulation behavior rather than comparative efficacy. The study duration for each subject extended from screening to final follow-up (48 hours post-dose), with an overall study period of seven days.

Ethical approval and regulatory compliance

The study protocol (HYPO-COR-2025-02, version 1.0, dated 10 July 2025), informed consent documents, and all relevant study materials were reviewed and approved by an Independent Ethics Committee (Goods Society for Ethical Research, Shahdara, Delhi) prior to study initiation (study approval number: GSER/2025/BMR/CL/240, dated 20 August, 2025). The study was conducted in accordance with the ethical principles of the Declaration of Helsinki [9], ICH E6 (R3) Good Clinical Practice (GCP) guidelines, Indian Council of Medical Research (ICMR) ethical guidelines, and applicable national regulatory requirements.

All participants provided written informed consent in a language they understood before undergoing any study-related procedures. The subject IDs were assigned to all volunteers to ensure the confidentiality and anonymity of the participants (C01, C04, C05, C06, C07, and C08). No confidential or personally identifiable information related to the participants has been disclosed in the manuscript.

Study population

Inclusion Criteria

The eligible participants were healthy male subjects between 18 and 45 years of age with a body mass index (BMI) ranging from 18.5 to 30 kg/m² and a body weight of at least 50 kg. All subjects were required to be in good general health, as confirmed by their medical history, physical examination, and laboratory investigations, with no clinically significant abnormalities. In addition, participants were required to demonstrate the ability and willingness to provide written informed consent prior to enrollment and the initiation of any study-related procedures.

Exclusion Criteria

The exclusion criteria included the presence of any clinically significant gastrointestinal, hepatic, renal, cardiovascular, neurological, endocrine, or hematological disorder that could potentially interfere with study outcomes. Individuals with a known hypersensitivity to vitamin B12 or any component of the investigational formulation, as well as those with active or recent gastrointestinal disorders that could affect drug absorption, such as peptic ulcer disease, were excluded. Additional exclusion criteria included clinically significant laboratory abnormalities, use of prescription or over-the-counter medications within 14 days before dosing, a history of drug abuse, excessive alcohol consumption (>10 units/week), or smoking more than 10 cigarettes/day. Furthermore, subjects who had participated in another clinical study within the preceding 90 days or had experienced significant radiation exposure within the previous 12 months were excluded from enrollment.

Investigational product

The investigational product was a bilayered oral tablet containing vitamin B12 (1500 µg) in sustained-release form. The formulation was designed as a dual-layer system where the upper layer was a placebo as Immediate-release, and the lower layer (matrix-controlled) showed sustained-release, designed to resist gastric disintegration and release drug in the intestinal environment. The tablets were manufactured by Corona Remedies Limited, Ahmedabad, Gujarat, India.

Radiolabelling technique

The investigational product was radiolabeled with ^99m^Tc using a validated surface-labeling protocol published by Sharma et al. [10]. This method ensured that the radiotracer remained associated with the dosage form without altering its structural integrity or release characteristics. Briefly, the radiotracer was reduced with a sufficient quantity of stannous chloride in aqueous and non-aqueous media. To enable differential visualization of the bilayer system, the upper placebo layer was labeled using an aqueous-phase tracer, representing rapid dispersion, and the lower layer of vitamin B12 was labeled using a non-aqueous-phase tracer, representing sustained-release behaviour. Further, the radiolabeling efficiency was determined using paper chromatographic techniques, which found 98.73 ± 1.09 and 97.25 ± 2.13, respectively, in aqueous and non-aqueous media. The administered radioactivity ranged from 100 to 500 µCi per tablet, which falls within accepted diagnostic exposure limits and is considered safe for human imaging studies.

Study procedures

All patients went through the screening procedure up to 21 days before drug administration. The screening procedure involved gathering information on demographics, medical history, physical examinations, vital signs (blood pressure, heart rate, body temperature), and laboratory tests like hematology, biochemistry, serology, and urinalysis. Only those patients who satisfied all the inclusion criteria but none of the exclusion criteria were selected for the study.

Subject Admission and Pre-dose Conditions

Subjects were admitted to the clinical facility approximately two hours prior to dosing following an overnight fast. Breath alcohol analysis and drug screening were performed at admission to confirm eligibility. Water intake was restricted from one hour before to one hour after dosing, except for the water administered with the investigational product. Subjects were required to abstain from alcohol, smoking, and grapefruit-containing products for at least 48 hours prior to dosing and throughout the study period.

Drug Administration

A single dose of the radiolabeled investigational product was administered orally with 240 mL of water under the direct supervision of a physician. Subjects were seated upright during administration and instructed to swallow the tablet whole without chewing, crushing, or dividing. Dosing compliance was verified by visual inspection of the oral cavity immediately after administration. Subjects remained in a semi-recumbent position for the first four hours post dose, with minimal movement allowed. Thereafter, normal ambulation was permitted.

Gamma scintigraphy imaging

Gamma scintigraphy was employed as a non-invasive imaging modality to dynamically track the in vivo transit, integrity, and disintegration behavior of the radiolabeled bilayered tablet within the gastrointestinal tract. Imaging was performed using a dual-head gamma camera system (Siemens Healthineers, Germany) equipped with a low-energy, high-resolution collimator optimized for detection of the 140 keV gamma emissions of 99mTc. Subjects were positioned in a standardized upright or semi-recumbent posture to maintain consistency across imaging time points and to minimize variability in gastrointestinal transit due to postural differences.

Immediately following oral administration of the radiolabeled tablet, baseline images were acquired within five minutes to confirm successful ingestion and localization of the dosage form within the stomach. This was followed by continuous dynamic imaging for the initial 15 minutes to capture rapid events such as the disintegration of the immediate-release upper layer and early gastric dispersion. Subsequent static images were obtained at predefined intervals of one, two, four, six, and eight hours post dose to monitor the progression of the dosage form through the gastrointestinal tract. Additional delayed imaging was performed when necessary to confirm colonic arrival of the tracer.

Image acquisition parameters, including acquisition time, energy window (typically ±10% around 140 keV), and matrix size, were kept constant throughout the study to ensure comparability of data. Care was taken to minimize motion artifacts by instructing subjects to remain still during image acquisition, and any deviations were documented. The imaging protocol was designed to capture both the rapid initial events associated with the immediate-release component and the delayed processes governing the sustained-release layer, thereby enabling comprehensive characterization of the biphasic release behavior.

Scintigraphic data analysis

Scintigraphic data were analyzed using a combination of qualitative visual assessment and semi-quantitative region-of-interest (ROI) analysis to derive key pharmacoscintigraphic parameters. Sequential images were reviewed to track the spatial distribution of radioactivity over time, allowing identification of the location of the radiolabeled tablet within anatomical regions such as the stomach, small intestine, and colon. For semi-quantitative analysis, ROI were manually delineated over the stomach, small intestine, and colon based on anatomical landmarks and distribution of tracer activity. Background correction was performed by selecting adjacent non-active regions to account for scatter and noise. Radioactivity counts within each ROI were expressed as a percentage of total administered activity, enabling normalization across subjects. Disintegration of the dosage form was defined by the transition from a discrete, well-defined radioactive hotspot (representing an intact tablet) to a diffuse pattern of activity, indicating dispersion of the formulation. The disintegration time of the immediate-release layer was identified during the early dynamic imaging phase, whereas disintegration of the sustained-release layer was determined during later time points when gradual spreading of tracer activity was observed in the intestinal region. Gastric residence time was calculated as the duration during which the majority (≥70%) of tracer activity remained within the gastric ROI, while gastric emptying time was defined as the time point at which a significant proportion of activity exited the stomach and appeared in the small intestine. Intestinal arrival time was determined when at least 10% of activity was detected beyond the pyloric region, and colon arrival time was defined by the appearance of tracer activity within the large intestine. Inter-subject variability was assessed by comparing temporal parameters across subjects.

Pharmacokinetic assessment

Pharmacokinetic evaluation was conducted to quantify systemic exposure to vitamin B12 following administration of the bilayered sustained-release formulation and to establish a temporal relationship between gastrointestinal release and absorption. Venous blood samples were collected at predefined intervals up to 48 hours post dose to capture both the early distribution phase and the prolonged absorption phase associated with sustained-release delivery. The sampling schedule was strategically designed to align with expected gastrointestinal transit events, particularly the delayed release of the sustained layer in the intestinal region. Baseline (pre-dose) samples were collected to determine endogenous vitamin B12 levels, followed by post-dose sampling at two, eight, 24, 36, and 48 hours. This time frame allowed for identification of the time to maximum concentration (Tmax), characterization of peak exposure (Cmax), and evaluation of sustained systemic levels over time. The concentration-time data were analyzed descriptively to determine key pharmacokinetic parameters, including Cmax and Tmax. Particular emphasis was placed on correlating the observed Tmax with scintigraphically determined intestinal disintegration time, thereby providing mechanistic insight into the relationship between site of release and systemic absorption. The sustained elevation of serum vitamin B12 levels over 48 hours was interpreted as evidence of prolonged absorption and extended systemic exposure attributable to the sustained-release component of the formulation. Although full non-compartmental pharmacokinetic modeling (e.g., area under the curve, half-life) was not performed due to the exploratory nature and limited sampling density, the available data were sufficient to support qualitative and semi-quantitative interpretation of the absorption profile. The integration of pharmacokinetic and scintigraphic data enabled a comprehensive evaluation of formulation performance [11].

Quantification of serum vitamin B12 concentrations was performed using chemiluminescence-validated analytical methods at an accredited clinical laboratory, ensuring compliance with established standards for bioanalytical accuracy and precision. Blood samples collected at each time point were processed under controlled conditions to preserve analyte stability. Following collection, samples were centrifuged within a specified time frame to separate plasma or serum, which was then stored at appropriate temperatures until analysis. The analytical method employed for vitamin B12 determination was based on established immunoassay techniques, which are widely used for clinical measurement of cobalamin levels.

Safety and tolerability assessment

Safety and tolerability of the investigational product were comprehensively evaluated throughout the study using a combination of clinical, laboratory, and observational assessments. All subjects who received the study drug were included in the safety analysis population. Safety monitoring was conducted from the time of informed consent until completion of the final study assessment at 48 hours post dose. Vital signs, including systolic and diastolic blood pressure, pulse rate, and body temperature, were measured at baseline, pre-dose, and at predefined intervals post dose (e.g., two, six, and eight hours), as well as at the end-of-study visit. Any clinically significant deviations from baseline values were documented and assessed for potential association with the investigational product. Clinical laboratory evaluations, including hematology, serum biochemistry, and urinalysis, were performed at screening and repeated at the end of the study. Hematological parameters included hemoglobin, hematocrit, total and differential leukocyte counts, and platelet count, while biochemical parameters encompassed liver function tests, including aspartate aminotransferase (AST), alanine aminotransferase (ALT), and bilirubin, renal function markers such as serum creatinine and blood urea nitrogen, and electrolytes. Urinalysis included assessment of physical and chemical parameters. All laboratory analyses were conducted at certified laboratories using validated methods, and results were reviewed for clinical significance by qualified medical personnel. Adverse events were actively monitored and recorded throughout the study period. Subjects were questioned at regular intervals regarding any symptoms or discomfort, and spontaneous reporting was also encouraged. Each reported adverse event was documented with details including onset, duration, severity (mild, moderate, or severe), outcome, and investigator-assessed causality in relation to the study drug (e.g., unrelated, unlikely, possible, probable). Serious adverse events, if any, were to be reported immediately to the ethics committee and sponsor. Particular attention was given to potential gastrointestinal adverse effects, given the nature of the formulation and its interaction with the gastrointestinal tract. Additionally, any signs of hypersensitivity or intolerance to vitamin B12 or excipients were closely monitored. Radiation exposure associated with the use of ^99m^Tc was within accepted diagnostic limits, and no radiation-related adverse effects were observed. Overall tolerability was assessed based on the absence or presence of clinically significant adverse events, changes in vital signs, and laboratory abnormalities. The investigational product was considered well tolerated if no serious or clinically meaningful adverse findings were identified. In this study, no adverse events were reported, and no clinically significant changes in vital signs or laboratory parameters were observed, indicating a favorable safety profile.

Statistical analysis

The statistical analysis of the obtained data was performed using GraphPad Prism version 10 (GraphPad Software, San Diego, CA, USA). Analysis of variance (ANOVA) was used for comparisons among the different groups. Results were presented as mean ± standard deviation (SD) and median.

## Results

Study population and disposition

A total of eight subjects were screened for eligibility, of whom six healthy male volunteers met all inclusion criteria and were enrolled in the study. All enrolled subjects completed the study as per protocol, and no dropouts or protocol deviations were reported. The mean age of the study population was 23.83 ± 6.37 years, with a mean body weight of 63.00 ± 6.78 kg, representing a relatively homogeneous group with low inter-subject variability. Baseline clinical and laboratory parameters were within normal limits for all participants, confirming the suitability of the study population for evaluation of formulation-driven effects without confounding physiological variability.

Pharmacoscintigraphic evaluation of gastrointestinal transit and disintegration

**Figure 1:**
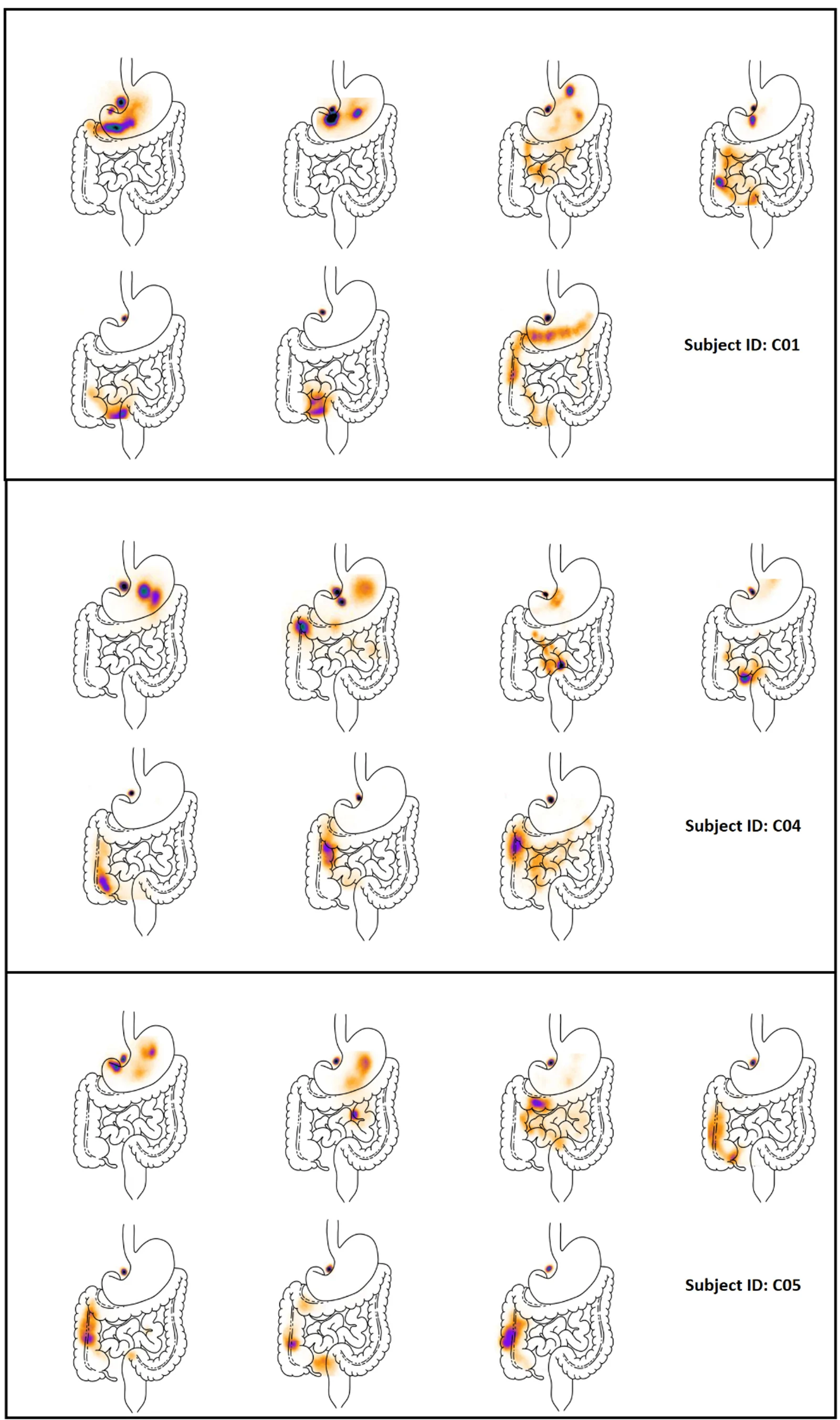
Representative gamma scintigraphy images The images show the in vivo gastrointestinal transit and distribution pattern of the radiolabeled formulation in healthy human volunteers (Subject IDs: C01, C04, and C05) at different post-administration time points. Images were captured using the gamma camera and processed on EvoCam software (Vision Engineering, Woking, UK). For a better understanding, the GIT template was superimposed on the obtained gamma images. The Subject IDs were assigned to all volunteers to ensure the confidentiality and anonymity of the participants. No confidential or personally identifiable information related to the participants has been disclosed in the manuscript.

**Figure 2:**
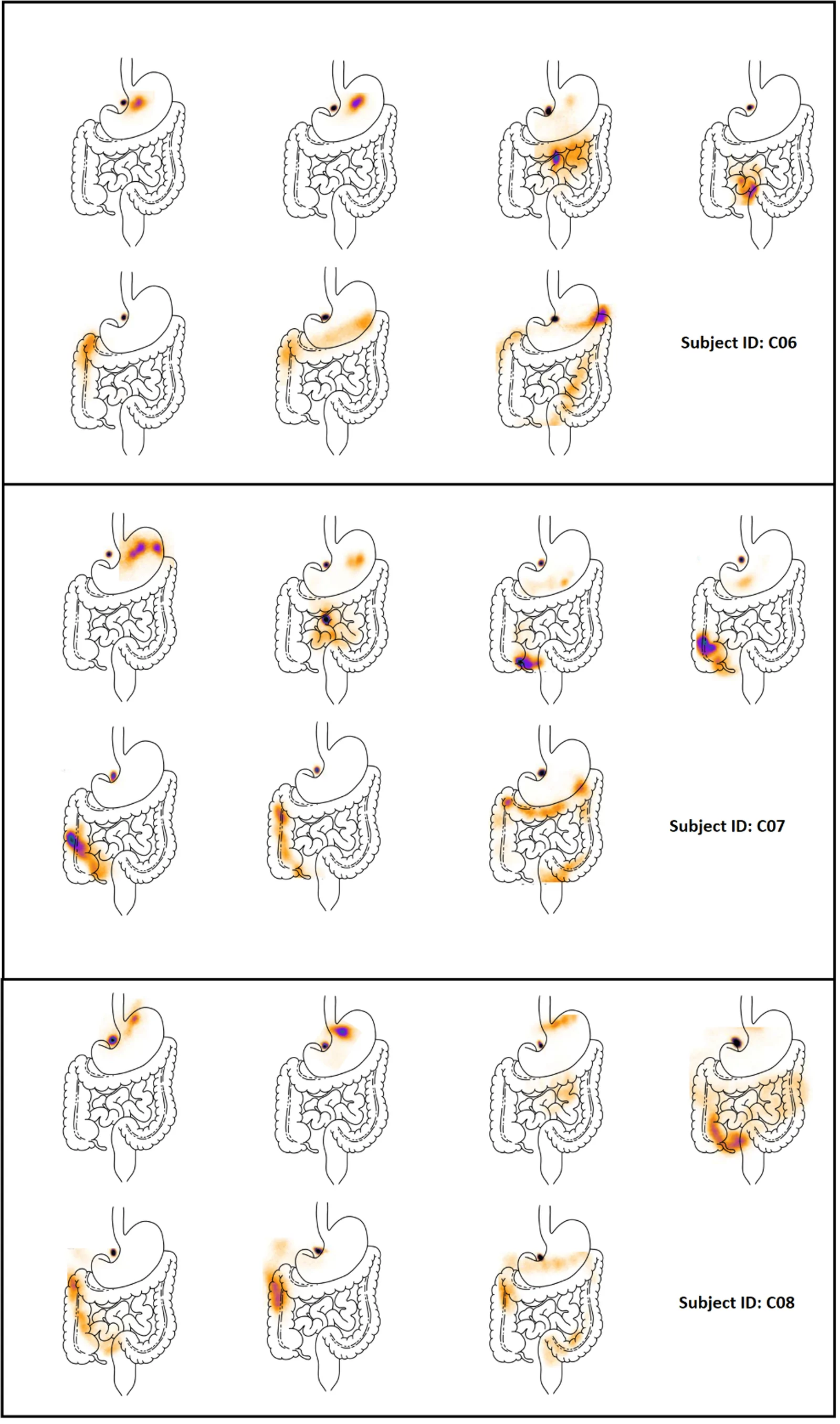
Representative gamma scintigraphy images The images show the in vivo gastrointestinal transit and distribution pattern of the radiolabeled formulation in healthy human volunteers (Subject IDs: C06, C07, and C08) at different post-administration time points. Images were captured using the gamma camera and processed on EvoCam software (Vision Engineering, Woking, UK). For a better understanding, the GIT template was superimposed on the obtained gamma images. The Subject IDs were assigned to all volunteers to ensure the confidentiality and anonymity of the participants. No confidential or personally identifiable information related to the participants has been disclosed in the manuscript.

Gamma scintigraphic imaging provided clear visualization of the in vivo behavior of the bilayered tablet and demonstrated a reproducible biphasic disintegration pattern across all six subjects (Figures 1, 2). Immediately following administration, the intact tablet was visualized as a discrete radiolabeled hotspot localized within the gastric region, confirming successful ingestion and initial gastric residence. During the early dynamic imaging phase, the immediate-release layer exhibited rapid disintegration within the stomach, with a mean disintegration time of 1.0 ± 0.0 minutes. This was characterized by a rapid transition from a well-defined hotspot to a diffuse distribution of radioactivity within the gastric contents, indicating prompt dispersion of the hydrophilic layer. The dispersed fraction was observed to undergo gastric emptying relatively quickly, with a mean emptying time of 0.7 ± 0.2 hours, suggesting minimal retention of the immediate-release component in the stomach.

In contrast, the sustained-release layer remained structurally intact during gastric residence, as evidenced by the persistence of a discrete radiolabeled focus in the stomach even after dispersion of the immediate-release layer. The intact sustained-release layer subsequently emptied from the stomach at a later time point, with a mean gastric emptying time of 120.00 ± 0.00 minutes. This sequential emptying behavior clearly demonstrated functional separation of the two layers and confirmed the intended biphasic design of the formulation. Following gastric emptying, the intact sustained-release layer progressed through the small intestine with minimal early dispersion. Onset of disintegration of the sustained-release layer was observed in the intestinal region at approximately four to six hours post dose. The gradual spreading of radioactivity within the intestinal tract indicated controlled release of the formulation contents, consistent with matrix-mediated sustained-release behavior.

Tracer activity was subsequently observed in the colon at approximately 336.00 ± 53.67 minutes in all subjects, indicating completion of small intestinal transit and confirming relatively consistent gastrointestinal motility across the study population. Overall, the scintigraphic findings demonstrated low inter-subject variability and a highly reproducible pattern of gastric disintegration, delayed intestinal release, and predictable gastrointestinal transit (Table 1).

**Table 1:** Gastrointestinal transit characteristics of the bilayer radiolabeled formulation in healthy human volunteers as determined by gamma scintigraphy. Data are presented as individual subject values along with the mean ± standard deviation and median. The Subject IDs were assigned to all volunteers to ensure the confidentiality and anonymity of the participants. No confidential or personally identifiable information related to the participants has been disclosed in the manuscript.

Subject Summary statistic	Disintegration time (minutes) of immediate layer	Disintegration time (minutes) of sustained-release layer	Gastric residence time (minutes)	Intestinal arrival time (minutes)	Intestinal residence time (minutes)	Colon arrival time (minutes)
C01	1	120	120	30	480	360
C04	1	90	120	30	480	360
C05	1	90	120	30	480	360
C06	1	90	120	30	360	240
C07	1	90	120	30	360	360
C08	1	90	120	30	360	360
Mean ± SD	1.00 ± 0.00	96.00 ± 13.42	120.00 ± 0.00	30.00 ± 0.00	432.00 ± 65.73	336.00 ± 53.67
Median (min-max)	1.0 (1.0 – 1.0)	90.0 (90.0 – 120.0)	120.0 (120.0 – 120.0)	30.0 (30.0 – 30.0)	480.0 (360.0 – 480.0)	360.0 (240.0 – 360.0)

Pharmacokinetic profile and systemic exposure

The serum concentration time profile of vitamin B12 following administration of the bilayered sustained-release formulation exhibited a delayed and sustained absorption pattern consistent with the observed scintigraphic behavior. Average baseline serum vitamin B12 levels in all volunteers were measured at 218.25 ± 57.75 pg/mL, reflecting endogenous physiological concentrations. Following administration, only a marginal increase in serum levels was observed at two hours (223.38 ± 55.88 pg/mL), indicating minimal early absorption during the gastric phase and proximal intestinal transit. This observation is consistent with the known physiology of vitamin B12 absorption, which primarily occurs in the distal ileum rather than in the stomach or upper small intestine. A pronounced increase in serum vitamin B12 concentration was observed at eight hours post dose, reaching a peak (Cmax) of 296.00 ± 94.65 pg/mL. The delayed time to maximum concentration (Tmax ≈ eight hours) corresponded closely with the scintigraphically determined timing of intestinal disintegration (approximately five hours) and subsequent transit to the distal small intestine, supporting the hypothesis that systemic absorption was primarily driven by release of the layered tablet in the intestinal region. Following the peak, serum vitamin B12 levels remained elevated above baseline at 24 hours (278.67 ± 66.14 pg/mL), 36 hours (259.86 ± 48.87 pg/mL), and 48 hours (262.00 ± 70.27 pg/mL), indicating prolonged systemic exposure. The persistence of elevated concentrations suggests sustained absorption and/or redistribution, consistent with the controlled-release characteristics of the formulation. Inter-subject variability in pharmacokinetic parameters was moderate but acceptable, with consistent trends observed across all participants (Table 2).

**Table 2:** Post-administration vitamin B12 concentrations Quantitative estimation of vitamin B12 at different post-administration time points in healthy human volunteers. Data are presented as individual participant values along with mean ± standard deviation and median (min–max). The Subject IDs were assigned to all volunteers to ensure the confidentiality and anonymity of the participants. No confidential or personally identifiable information related to the participants has been disclosed in the manuscript.

Participant/Summary statistic	0 minutes	2 hours	8 hours	24 hours	36 hours	48 hours
C01	174	166	206	193	189	177
C04	255	304	330	302	279	294
C05	138	143	209	214	202	174
C06	298	260	317	305	298	317
C07	225	272	496	374	324	362
C08	171	197	258	284	263	271
Mean ± SD	218.25 ± 57.75	223.38 ± 55.88	296.00 ± 94.65	278.67 ± 66.14	259.86 ± 48.87	262.00 ± 70.27
Median (min-max)	212.0 (138.0 – 298.0)	222.5 (143.0 – 304.0)	287.0 (206.0 – 496.0)	293.0 (193.0 – 374.0)	264.0 (189.0 – 324.0)	271.0 (174.0 – 362.0)

Correlation between scintigraphic and pharmacokinetic findings

A strong temporal correlation was observed between scintigraphic parameters and pharmacokinetic outcomes, providing mechanistic validation of the formulation performance. The delayed onset of systemic exposure coincided with the timing of intestinal disintegration of the sustained-release layer, suggesting that most absorption occurred after release in the small intestine. The observed Tmax at eight hours closely matched the expected transit time to the distal ileum, reinforcing the concept of site-specific absorption. This integrated analysis confirmed that the developed formulation successfully aligned with the gastric disintegration from systemic absorption and achieved targeted delivery of vitamin B12 to the physiological absorption site. The concordance between imaging and pharmacokinetic data provides strong evidence supporting the functional design of the formulation.

Safety and tolerability

The investigational product was well tolerated by all subjects, with no adverse events reported during the study period. No clinically significant changes were observed in vital signs, including blood pressure, pulse rate, and body temperature, at any time point following administration.

Clinical laboratory parameters, including hematology, serum biochemistry, and urinalysis, remained within normal limits throughout the study, and no clinically relevant abnormalities were detected at the end-of-study assessment. There were no signs of gastrointestinal intolerance, hypersensitivity reactions, or any other safety concerns related to the investigational product (Data are provided in Appendix A). Overall, the safety findings indicate that the bilayered sustained-release formulation is safe and well tolerated under the conditions of this study. The combined pharmacoscintigraphic and pharmacokinetic results clearly demonstrate that the bilayered vitamin B12 sustained-release tablet achieves its intended design objectives. The formulation exhibits delayed gastric emptying and intestinal release of the sustained-release layer, temporal alignment of intestinal disintegration with peak systemic exposure, and sustained plasma levels up to 48 hours. These findings provide direct in vivo evidence of site-specific drug delivery and controlled release, supporting the formulation’s potential to improve the pharmacokinetic behaviour of vitamin B12.

## Discussion

The present study provides integrated pharmacoscintigraphic and pharmacokinetic evidence supporting the in vivo performance of a bilayered vitamin B12 sustained-release formulation. By combining real-time imaging of gastrointestinal transit with systemic exposure profiling, this investigation offers mechanistic insights into how formulation design governs spatial and temporal drug release, ultimately influencing absorption. The findings demonstrate that the formulation successfully achieves a delayed intestinal release of the sustained-release component. One of the most notable observations was the rapid disintegration of the upper layer within approximately two minutes of administration, followed by its prompt gastric emptying. This finding is physiologically consistent with the known absorption characteristics of vitamin B12, which relies predominantly on intrinsic factor-mediated uptake in the terminal ileum. The absence of early systemic exposure reinforces the concept that gastric or proximal intestinal release alone is insufficient for meaningful absorption of vitamin B12, thereby highlighting the importance of site-specific delivery [12]. In contrast, the sustained-release lower layer exhibited delayed gastric emptying and remained intact during its transit through the stomach. This behavior is critical, as premature disintegration in the gastric environment would compromise the ability of the formulation to deliver the drug to the distal intestine. The observed gastric emptying time of approximately 120.00 ± 0.00 minutes for the intact lower layer falls within the expected physiological range for solid dosage forms under fasting conditions, suggesting that the formulation does not significantly alter normal gastric motility. More importantly, the preservation of structural integrity during gastric residence confirms the robustness of the matrix system employed in the sustained-release layer. The onset of disintegration of the sustained-release layer was observed in the intestinal region at approximately four to six hours post-dose, with complete dispersion occurring shortly thereafter. This delayed-release profile is particularly significant in the context of vitamin B12 absorption, as it aligns closely with the transit time required to reach the distal small intestine [3,13]. The temporal correspondence between intestinal disintegration and the subsequent rise in serum vitamin B12 levels provides strong evidence that the formulation effectively targets the physiological absorption window. The peak plasma concentration observed at eight hours further supports this interpretation, as it reflects the time required for drug release, absorption, and systemic distribution following ileal uptake. The sustained elevation of serum vitamin B12 levels up to 48 hours post dose suggests prolonged absorption and/or redistribution, consistent with the controlled-release characteristics of the formulation. This extended exposure profile may offer clinical advantages, including reduced dosing frequency and improved patient adherence, particularly in populations requiring long-term supplementation. Furthermore, the absence of a sharp early peak in serum concentration indicates that the formulation minimizes burst release, thereby reducing the risk of transient supraphysiological exposure [14].

A key strength of this study lies in the integration of pharmacoscintigraphy with pharmacokinetic analysis, which allows for direct correlation between the physical behavior of the dosage form and its systemic effects. Traditional pharmacokinetic studies alone are often insufficient to elucidate the underlying mechanisms of drug release and absorption, particularly for modified-release formulations. In contrast, pharmacoscintigraphy provides spatial and temporal resolution of dosage form transit and disintegration, enabling a more comprehensive understanding of formulation performance [15]. The strong concordance observed between scintigraphic and pharmacokinetic data in this study underscores the value of this combined approach in the evaluation of site-specific drug delivery systems. The low inter-subject variability observed in both scintigraphic and pharmacokinetic parameters further strengthens the reliability of the findings. Consistent gastric emptying times, intestinal transit patterns, and delayed Tmax values across subjects indicate that the formulation performs reproducibly under physiological conditions. This is an important consideration for clinical translation, as variability in gastrointestinal transit can often lead to unpredictable drug release and absorption profiles. The ability of the formulation to maintain consistent performance despite inherent physiological variability suggests a robust design.

The major limitation of the current study is the limited number of human volunteers. Future experiments with increased numbers of participants and different demographics such as men, women, elderly people, and individuals suffering from vitamin B12 deficiency would allow for wider clinical application of the formulation. Experiments comparing the performance of the formulation to that of immediate-release formulations will be valuable in determining the performance of the developed sustained-release formulation.

## Conclusions

This study illustrated the successful in vivo application of a sustained-release tablet intended for site-specific delivery via the oral route. The results from pharmacoscintigraphy and pharmacokinetic analysis provided clear evidence of the tablet during the gastric phase of passage through the body and subsequent drug release during the intestinal phase. There was a high level of correlation between the data obtained from both analyses, particularly in the delayed maximum peak plasma concentration at 48 hours, indicating that the sustained-release formulation is an ideal approach to improve oral delivery. The developed formulation was effective with little intersubject variability, a good safety profile, and was well tolerated. Radiolabeling of the formulation using ^99m^Tc was found to be safe and effective for in vivo assessment. This study clearly shows compelling evidence supporting sustained-release tablets to enhance oral delivery and improve site-specific activity.

## Appendices

Appendix A

Pre-dosing and Post-dosing Parameters

https://1drv.ms/f/c/1bb2894b59c15ae4/IgCQv7T-tQaYRr2TK1tuKR34AfaCyOXcurgMhUQwrXbZIT0?e=03rNWn
